# One anastomosis gastric bypass as a one-stage bariatric surgical procedure in patients with BMI ≥ 50 kg/m^2^

**DOI:** 10.1038/s41598-022-14485-3

**Published:** 2022-06-22

**Authors:** Mohammad Kermansaravi, Seyed Nooredin Daryabari, Reza Karami, Seyed Amin Setaredan, Rohollah Valizadeh, Samaneh Rokhgireh, Abdolreza Pazouki

**Affiliations:** 1 Department of Surgery, Minimally Invasive Surgery Research Center, Division of Minimally Invasive and Bariatric Surgery, Rasool-e Akram Hospital, School of Medicine, Iran University of Medical Sciences, Tehran, Iran; 2 Center of Excellence of European Branch of International Federation for Surgery of Obesity, Hazrat-e Rasool Hospital, Tehran, Iran; 3 Minimally Invasive Surgery Research Center, Iran University of Medical Sciences, Tehran, Iran; 4 Department of Epidemiology, Student Research Committee, School of Public Health, Iran University of Medical Science, Tehran, Iran; 5 Endometriosis Research Center, School of Medicine, Iran University of Medical Sciences, Tehran, Iran

**Keywords:** Endocrinology, Gastroenterology

## Abstract

In patients with BMI ≥ 50 kg/m^2^, it is difficult to select an appropriate procedure that can lead to optimum results. This study aims to evaluate mid-term weight loss outcomes in patients with BMI ≥ 50 kg/m^2^ following one anastomosis gastric bypass (OAGB) as a one-stage procedure. A prospective study was conducted on patients with BMI ≥ 50 kg/m^2^, aged 18 years and above who had undergone primary OAGB from January 2016 to February 2019 with at least two years follow-ups. A total of 197 patients with BMI ≥ 50 kg/m^2^ had underwent OAGB. The mean age was 38 years and the mean pre-operative BMI was 53.7 kg/m^2^. Mean EWL% were 63.7%, 67.8% and 66.2% at one, two and five years after OAGB respectively. The highest level of EWL% was 68.4%, which was achieved in the 18th month following OAGB. OAGB can be performed safely in patients with BMI ≥ 50 kg/m^2^ as a one-stage procedure with acceptable weight loss outcomes and remission of obesity associated medical problems.

## Introduction

Obesity is one of the leading medical problems worldwide that may need surgical bariatric interventions in morbidly obese patients.

In patients with BMI ≥ 50 kg/m^2^, it is difficult to select an appropriate procedure that can lead to optimum results. BMI ≥ 50 kg/m^2^ may be a risk factor for more challenging bariatric procedures because of the patient’s physical characteristics^1^ and may lead to insufficient weight loss and weight regain after the surgery. Some studies recommend more potent procedures such as biliopancreatic diversion (BPD) with or without a duodenal switch (BPD-DS), one anastomosis gastric bypass (OAGB) and Roux-en Y gastric bypass (RYGB), while others recommend two-stage procedures with SG as the first stage, followed by either RYGB, OAGB, or BPD-DS^2,3^. There is no high-grade evidence regarding the most appropriate procedure for this class of obesity to reach maximal efficacy in weight loss outcomes and remission of obesity associated medical problems with low complication rates. Although in a recently published experts consensus, more than 90% of experts believed that OAGB is an appropriate one-stage bariatric surgical procedure in patients with BMI ≥ 50 kg/m^2^^4^. There are few published paper about the efficacy of one-stage OAGB in patients with BMI ≥ 50 kg/m^2^^5–7^ and need to be supported by more studies.

This study aims to evaluate mid-term outcomes of one-stage OAGB in patients with BMI ≥ 50 kg/m^2^ on weight loss outcomes and remission of obesity associated medical problems.

## Materials and methods

This prospective study was conducted on patients with severe obesity and BMI ≥ 50 kg/m^2^ aged 18 years and above who had undergone primary OAGB from January 2016 to February 2019 with at least 2 years follow-ups at the Rasool-e Akram Hospital, an accredited Center of Excellence of the European Branch of the International Federation for Surgery of Obesity (IFSO).

Exclusion criteria consisted revisional/conversional OAGB, lost to follow-up and pregnancy during mid-term follow-ups. According to the time of surgery, only 83 of 196 patients (43.8%) completed their 5-year follow-up.

All the patients were screened before the surgery by a multidisciplinary team consisting of an endocrinologist, a bariatric surgeon, a bariatrician physician, a gastroenterologist, a sports medicine specialist, a nutritionist and a psychiatrist/psychologist.

We routinely performed preoperative esophagogastroduodenoscopy (EGD) and biopsy to evaluate the presence of gastroesophageal reflux disease (GERD), hiatal hernia and *Helicobacter pylori* (HP). In patients with positive HP, eradication were done before OAGB.

All the patients had follow-ups at 10 days, and 1, 3, 6, 9, 12 and then annually after OAGB.

Weight loss outcomes were defined as percent excess weight loss (%EWL) and percent of total weight loss (TWL %)

$$\begin{gathered} \% EWL:\left[ {\left( {\text{Initial Weight}} \right) \, {-} \, \left( {{\text{Post}} - {\text{Op Weight}}} \right)} \right]/ \, \left[ {\left( {\text{Initial Weight}} \right) \, {-} \, \left( {\text{Ideal Weight}} \right)} \right] \times { 1}00 \hfill \\ \% TWL:\left[ {\left( {\text{Initial Weight}} \right) \, {-} \, \left( {{\text{Post}} - {\text{Op Weight}}} \right)} \right]/ \, \left[ {\left( {\text{Initial Weight}} \right)} \right] \, \times { 1}00. \hfill \\ \end{gathered}$$

The major complications were defined as any complication that resulted in a prolonged hospital stay (beyond 7 days), re-intervention or reoperation, such as anastomotic leak requiring reoperation, a venous thrombotic event (VTE), and gastrointestinal hemorrhage^8^.

Remission of obesity associated medical problems including type-2 diabetes mellitus (T2DM), arterial hypertension (HTN), dyslipidemia (DLP), obstructive sleep apnea (OSA) and GERD, before and after OAGB at the defined follow-up intervals were according to the ASMBS standardized outcomes reporting^8^ as following:

### Complete remission of T2DM

HbA1c < 6% or FBG < 100 mg/dl in the absence of antidiabetic medications.

### Improvement of T2DM

Statistically significant reductions in HbA1c and FBG not meeting the criteria for remission or discontinuing insulin or one oral agent or a 50% reduction in dosage.

### Complete remission of HTN

BP < 120/80 with no antihypertensive medications.

### Improvement of HTN

Decrease in dosage or number of antihypertensive medications or a decrease in systolic or diastolic blood pressure with the same medications.

### Remission of DLP

A normal lipid panel off-medication.

### Complete remission of OSA

Subjective method based on the patient’s discontinuance of CPAP or sleeping better on lower CPAP settings.

### Complete resolution of GERD

Subjective method according to absence of symptoms and no medication use.

The study protocol was approved by the ethics committee of the Iran University of Medical Sciences under this number: IR.IUMS.REC.1399.1198.

### Surgical procedures

OAGB was performed with five trocars laparoscopic technique in French position. The gastric pouch was constructed on a 36-Fr tube along the lesser curvature with 60 mm length linear staplers (Endo-GIA), beginning from the distal part of the crow’s foot to the His angle. Then, gastro-jejunostomy was performed with a 45 mm length linear stapler (Endo-GIA) at the anastomotic length of 40 mm in the posterior wall of the pouch, side to side with the jejunum, with a biliopancreatic limb (BPL) of 200 cm for all the patients. The enterotomies were closed with one-layer absorbable suture (PDS 2-0). Finally, after obtaining a negative air leak test, a drain was placed. All patents received 5000 IU Heparin/SC before starting the surgery that be continued every 8 h for two-weeks after OAGB.

On the first postoperative day, after the methylene blue leak test and clear liquid tolerance, the drain was removed and the patient was discharged in accordance with the Enhanced Recovery after Bariatric Surgery (ERAS) protocol.

### Data collection

Data on the patients’ age, sex, weight, BMI and obesity associated medical problems were assessed preoperatively. All pre-operative, operative and post-operative data including lab data, complications and follow-ups were registered in Iran National Obesity Surgery Database (INOSD)^9^, which is a web-based national registry database.

### Statistical analysis

The mean, percentage and 95% confidence interval (CI) were reported for the description of the data. Repeated measurements were used to assess the trend of changes in weight, BMI and EWL overall. All the analyses were carried out in SPSS version 25.0 (Chicago, Illinois, USA).

### Ethical approval statement

All procedures performed in the study involving human participants were in accordance with the ethical standards of the institutional and/or national research committee and with the 1964 Helsinki declaration and its later amendments or comparable ethical standards.

### Informed consent statement

Informed consent was obtained from the participants included in the study.

## Results

A total of 197 patients with BMI ≥ 50 kg/m^2^ had underwent OAGB. Table 1 shows the basic demographics and obesity related medical problems in the patients. The mean age was 38 years and the mean pre-operative BMI was 53.7 kg/m^2^. There was one mortality due to venous thromboembolism (VTE) two weeks after surgery. There was no other major complications in peri-operative period.

**Table 1:** Basic demographics of patients undergoing one anastomosis gastric bypass.

No. of cases		197	
Males		63 (30.9%)	
Age (mean and range)		38 (18.2–69.3)	
Weight (kg) (mean and range)		151.6 (112.1–257)	
BMI (kg/m^2^) (median and range)		53.7 (50–114.6)	
Type 2 diabetes mellitus		35 (17.8%)	
Dyslipidemia		48 (24.4%)	
Hypothyroidism		37 (18.8%)	
Hypertension		33 (16.8%)	
Sleep apnea		41 (20.8%)	
GERD		24 (12.1%)	
Urine stress incontinency		44 (22.3%)	

Minor complications were present as extra-luminal bleeding in six patients and two wound infections, which all were managed conservatively.

**Figure 1:**
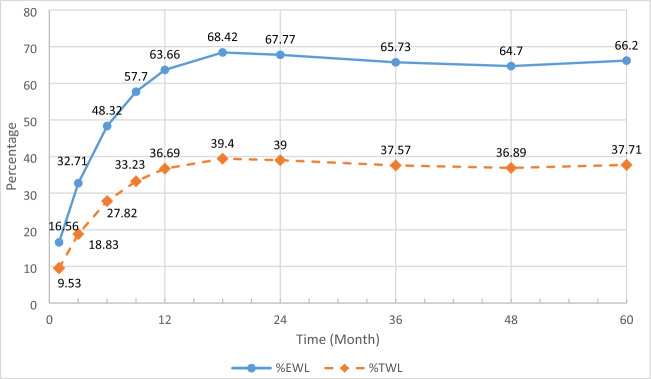
Percent excess weight loss (%EWL) and percent of total weight loss (%TWL) trend in patients with BMI ≥ 50 kg/m^2^ undergoing OAGB.

Table 2 shows the TWL% and EWL% at 6 months, 1 year, 2 and 5 years after OAGB. Mean EWL% were 63.7%, 67.8% and 66.2% at 1, 2 and 5 years after OAGB respectively. The highest level of EWL% was 68.4%, which was achieved in the 18th month following OAGB.^10^. Figure 1, shows the TWL% and EWL% trends during 60 months follow-ups.

**Table 2:** Weight loss outcomes in patients undergoing one anastomosis gastric bypass.

	Total patients	Mean (95% CI)
TWL at 6 months	196	42.4 (19.6–80)
% EWL at 6 months		48.3 (27.2–80.7)
% TWL at 6 months		27.8 (15.7–43.6)
TWL at 12 months	196	55.9 (29.6–100)
% EWL at 12 months		63.7 (38.1–89.5)
% TWL at 12 months		36.7 (23.2–56.8)
TWL at 2 years	191	59.4 (4.1–127)
% EWL at 2 years		67.8 (5.1–110)
% TWL at 2 years		39 (2.8–61.1)
% EWL at 3 years	172	65.7 (0.6–98.2)
% TWL at 3 years		37.6 (0.3–61.1)
% EWL at 5 years	83	66.2 (29.3–98.9)
% TWL at 5 years		37.7 (17.9–62.9)

The patients also had acceptable results in their obesity associated medical problems remission (Table 3).

**Table 3:** Obesity associated medical problems remission in super-obese patients undergoing one anastomosis gastric bypass.

Obesity associated medical problems (remission)	Total	N (%)	Mean time to remission (range) (month)
Type 2 diabetes mellitus	35	33 (94.29)	4.5 (0.97–13.2)
Dyslipidemia	48	35 (72.9)	6.4 (0.73–34)
Hypothyroidism	37	13 (35.1)	9.1 (1.07–44.73)
Hypertension	33	30 (90.9)	7.3 (0.93–48.67)
Obstructive sleep apnea	71	71 (100)	3.7 (1.02–5.3)
GERD	24	22 (91.6%)	8.2 (4.7–12.3)
Urine stress incontinency	44	41 (93.1%)	12.7 (9.8–17.4)

## Discussion

Bariatric surgery in patients with BMI ≥ 50 kg/m^2^ may have higher risk compare to lower BMIs ranges^11^ and several studies have questioned the safety and perioperative outcomes of bariatric surgery in patients with BMI ≥ 50 kg/m^2^ and produced discordant results^5^. The management of patients with BMI ≥ 50 kg/m^2^ is challenging because of the associated medical problems, patients' characteristics such as large liver size, resulting in decreased workspace^1,12^.

According to the first survey of bariatric surgeons about patients with BMI ≥ 50 kg/m^2^, about 53% of participants recommended one-stage procedure and 47% of them recommended a two-stage approach with SG as the first step for these patients^13^. Also, some authors have recommended two-stage procedures in patients with BMI ≥ 50 kg/m^2^, including sleeve gastrectomy (SG) as a first-stage procedure and RYGB or BPD-DS as the second-stage procedure in order to decrease complications in these patients^14,15^.

Some studies have demonstrated the effectiveness of OAGB on weight loss and obesity associated medical problems as a primary bariatric procedure. A multicenter study showed an EWL of 77% in a five-year follow-up after OAGB^16^. In a systematic review by Parmar et al., the mean %EWL at one year, up to two years and five years after OAGB was 67.7%, 71.6%, and 90.75%, respectively^17^, which are comparable with the present findings, as the mean EWL% was 63.7% and 67.8% at the 1st and 2nd year of the follow-up in this study. Our results show better EWL% compare to a similar study that performed SG in super obese patients with one year mean EWL% of 55.5 ± 16.8% that 24 of 41 their cases underwent second staged Roux-en-Y gastric bypass after initial SG^18^. Bhandari et al. showed that OAGB has significantly lower rates of 3-years weight loss failure compare to SG and RYGB in patients with BMI ≥ 50 kg/m^2^^5^. Another study reported the same results in OAGB superiority in weight loss outcomes and prevention of 5-year weight regain in comparison to SG and RYGB^6^. In a study, SG lead to EWL% of 51% at 3-year follow-up in patients in this class of obesity^19^. Plamper et al. showed significantly better one-year weight loss and lower complication rate after OAGB compare to SG in patients with BMI ≥ 50 kg/m^2^^20^. Parmar et al. showed that OAGB has a significantly superiority in two-year weight loss outcomes with less complication rate compare to RYGB in patients with BMI ≥ 50 kg/m^2^^21^. A recently published study showed that OAGB not only has better weight loss outcomes, but also has shorter operation time and hospital stay and lower complication rates in comparison to SG in patients with BMI > 60 kg/m^2^^22^. Singla et al. had the same results in their study in this class of obesity. They found that OAGB has significantly superior efficacy in one-year weight loss outcomes with lower complications compare to SG^23^. These findings support the superiority of OAGB in weight loss outcomes in patients with BMI ≥ 50 kg/m^2^ compare to SG and RYGB.

Our study shows that OAGB can be done as a safe and low complication procedure in this group of patients. There was only one mortality (0.5%) due to venous thromboembolism (VTE) two weeks after surgery, without any more major complications in peri-operative period and during follow-ups that show the safety of OAGB in patients with BMI ≥ 50 kg/m^2^.

Liagre et al.^7^ in a study on 245 patients with BMI ≥ 50 kg/m^2^ who underwent OAGB with a BPL of 150 cm, showed that OAGB is an effective method with low complication rates(5.7%) and no mortality. In their study, only 0.8% of patients needed to be underwent for second bariatric procedure due to weight loss failure. Another study by Soong et al. showed that OAGB has better five-year weight loss outcomes in comparison to SG and RYGB in patients with BMI ≥ 50 kg/m^2^ as a one-stage bariatric procedure with low overall complications and protein-malnutrition rates (8.5% and 4.1%), that was less than RYGB(17.7% and 6.5%)^6^. However, Diaz-Tobarra et al.^3^ reported that both one and two-stage bariatric procedures result in good long-term outcomes in patients with BMI ≥ 50 kg/m^2^ and revealed that the patients who had underwent one-stage RYGB even showed a significantly lower BMI at the five-year follow-up. Shorter operating time is another advantages of OAGB compare to procedures such as RYGB that are more complex in this class of obesity. OAGB can reduce the need for second weight loss procedures in patients with BMI ≥ 50 kg/m^2^ in comparison with SG^24^. Contrary SG can be a negative factor in weight loss outcomes in patients with BMI ≥ 50 kg/m^2^ compare to gastric bypass that needs second-stage bariatric procedure^25^.

Our study also showed the efficacy of OAGB in remission of obesity associated medical problems including T2DM, DLP, HTN, OSA and GERD in 94.2%, 72.9%, 90.9%, 100% and 91.6% of patients with BMI ≥ 50 kg/m^2^ respectively. Liagre et al.^7^ showed evolution of T2DM, DLP, HTN and OSA after OAGB in 92%, 98%, 76% and 97% of patients with BMI ≥ 50 kg/m^2^ respectively that were nearly similar to our findings. Although the efficacy of OAGB in remission of T2DM was as the same as SG in some studies^6,22,23^, OAGB has significant superiority in remission of other obesity associated medical problems in patients with BMI ≥ 50 kg/m2. Soong et al. showed the superior efficacy of OAGB in remission of HTN and DLP compare to SG, despite the more incidence of anemia and hypoalbuminemia in OAGB group^6^.

It has been shown that OAGB with a 250 cm BPL in presence of total bowel length more than 600 cm has significant efficacy on weight loss and remission of associated medical problems without any significant nutritional deficiency in patients with severe obesity and BMI ≥ 50 kg/m2^26^. The total bowel length measurement to have at least 400 cm common channel can decrease the incidence of malnutrition after OAGB^27^.

This study has some main limitations. The first limitation is our relatively low sample size and prospective observational study without any randomization trial. The second one was the single-center design of our study. Another limitation is the mid-term to 5-year follow-up time and more than half of patients did not reach to their 5-year follow-up according to the time of surgery. The last limitation was OAGB with a constant 200 cm BPL without measuring of total small bowel length.

## Conclusion

In conclusion, OAGB can be performed safely in patients with BMI ≥ 50 kg/m^2^, as a one-stage bariatric procedure without a significant complication rate. OAGB can leads to acceptable weight loss outcomes and remission of obesity-associated medical problems. Shorter operative time is another advantage of OAGB in high-risk obese patients. Nevertheless, further evaluations in larger randomized clinical trials with longer follow-ups are required to ascertain this conclusion (supplementary information).

## Supplementary Information

Supplementary Information.

## Data Availability

The data that support the findings of this study are available from Iran National Obesity Surgery Database (INOSD) but restrictions apply to the availability of these data, which were used under license for the current study, and so are not publicly available. Data are however available from the authors upon reasonable request and with permission of INOSD.
